# Structural Mechanism of S-Adenosyl Methionine Binding to Catechol O-Methyltransferase

**DOI:** 10.1371/journal.pone.0024287

**Published:** 2011-08-31

**Authors:** Douglas Tsao, Luda Diatchenko, Nikolay V. Dokholyan

**Affiliations:** 1 Department of Chemistry, University of North Carolina, Chapel Hill, North Carolina, United States of America; 2 Center for Neurosensory Disorders, School of Dentistry, University of North Carolina, Chapel Hill, North Carolina, United States of America; 3 Department of Biochemistry and Biophysics, School of Medicine, University of North Carolina, Chapel Hill, North Carolina, United States of America; University of South Florida College of Medicine, United States of America

## Abstract

Methyltransferases possess a homologous domain that requires both a divalent metal cation and S-adenosyl-L-methionine (SAM) to catalyze its reactions. The kinetics of several methyltransferases has been well characterized; however, the details regarding their structural mechanisms have remained unclear to date. Using catechol O-methyltransferase (COMT) as a model, we perform discrete molecular dynamics and computational docking simulations to elucidate the initial stages of cofactor binding. We find that COMT binds SAM via an induced-fit mechanism, where SAM adopts a different docking pose in the absence of metal and substrate in comparison to the holoenzyme. Flexible modeling of the active site side-chains is essential for observing the lowest energy state in the apoenzyme; rigid docking tools are unable to recapitulate the pose unless the appropriate side-chain conformations are given *a priori*. From our docking results, we hypothesize that the metal reorients SAM in a conformation suitable for donating its methyl substituent to the recipient ligand. The proposed mechanism enables a general understanding of how divalent metal cations contribute to methyltransferase function.

## Introduction

Catechol O-methyltransferase (COMT) is a metalloenzyme that metabolizes biologically active catechol-containing structures by methylation of a single hydroxyl group [1]. A number of neurotransmitters contain a catecholamine moiety and are deactivated by COMT. Activity of COMT is thus correlated with many critical biological functions including cognition [2], stress response [3], and pain sensitivity [4].

Crystallographic and enzymatic studies of COMT have given significant insight to its mode of activity [5], [6]. Its ligands bind in a sequential manner. The cofactor S-adenosyl-L-methionine (SAM), responsible for donating the methyl group to the catechol, initially binds to the enzyme. This complex then binds a divalent metal cation through coordination bonds to several acidic residues and a single water molecule in the active site. Additional residues then bind to the catechol substrate, and the metal coordinates to the hydroxyl group inside the active site. The order in which each ligand binds to COMT is vital; if the metal binds first, then the SAM cofactor is unable to access its binding site. Similarly, if the catechol binds first, both the metal and SAM are unable to access their respective binding sites. Although the general kinetic mechanism for COMT substrate binding is known, the structural details that govern binding have yet to be identified. Current crystal structures of COMT are bound to SAM, Mg^2+^, and an inhibitor. Using computational docking tools (Experimental Section; [7], [8], we dock SAM to an apo-COMT protein to determine the initial binding poses. We also perform docking simulations with SAM inside a COMT•metal•catechol complex (holo-COMT) and compare to the crystal structure. From our results, we find that SAM binds to COMT via induced fit and also hypothesize that the metal cation is critical for aligning SAM in a conformation suitable for catalysis with the substrate. Here we propose a mechanism whereby SAM initially binds to COMT, and upon metal binding, subsequently reorients itself for the enzyme to accommodate the substrate and facilitate methyl transfer.

## Methods

### Discrete Molecular Dynamics Simulation of COMT

We generate a conformation of COMT in the absence of ligands by performing discrete molecular dynamics simulations (DMD). Traditional molecular dynamics simulate the motions of particles by solving Newton's equations of motion for a defined system using an integration algorithm. In DMD, simulations proceed according to the conservation laws of energy, momentum, and angular momentum and are evaluated as a series of two-body interactions. The efficiency of the engine is based on an algorithm that searches through an event table, where velocities are only modified as necessary. Here we classify an event as the instance in which two particles are within a defined interaction range as defined by their potential. The potentials used in DMD are discretized to accommodate the discontinuous nature of the simulations. Further details of the DMD algorithm can be found elsewhere [9].

To observe any potentially major conformational changes due to the absence of ligands, we performed DMD simulations of COMT at a temperature of 0.2 ε/k_B_ (using a Berendsen thermostat) for 1×10^6^ time units to ensure final equilibration. Further minimization was performed in order to remove any potential steric clashes and obtain the final structure.

### Generation of Poses using MedusaDock

We employ the MedusaDock package to generate possible ligand conformations within the protein active site, and utilize the MedusaScore package to evaluate each conformation generated by MedusaDock [7], [8]. MedusaDock enables flexible docking of both the ligand and the side-chain amino acids of the protein. We perform all ligand docking simulations using the crystal structure of human COMT (PDB: 3BWM) [5].

Prior to docking S-adenosyl methionine (SAM) to apo-COMT, we stripped all crystallized ligands bound to COMT. Crystallographic waters that were in the active site were retained for our docking simulations. Protein residues were protonated accordingly. We define a 10×10×10 Å box for our docking simulations. Within this box, the amino acid side-chains from the protein are able to move.

We performed 200 docking simulations, where each simulation began with a different seed number and each conformation generated from a simulation was subsequently minimized. All conformations were then ranked according to their free energy value. The lowest energy structure is determined to be the native pose.

To model the entire complex with SAM, Mg^2+^, and catechol bound (holo-COMT), we add several constraints and make several modifications. First, we replace Mg^2+^ with Zn^2+^ since there are no Mg^2+^ parameters previously defined in the MedusaScore force field. However, previous experiments suggest that Zn^2+^ is a suitable alternative for Mg^2+^ as it is ∼80% as effective [10]. The general mechanism is that the metal divalent ion displaces the monovalent cationic amine group and acts as a steric block for the rest of the methionine side-chain. We place constraints to fix the position of the metal and catechol as found in the crystal structure, while we flexibly dock SAM into the active site. We find that the lowest energy pose recapitulates the crystal structure, with a heavy atom root mean square deviation of 0.68 Å.

### Generation of Poses using Glide

Two sets of docking simulations were performed with Glide (Maestro package version 9.1 from Schrödinger, LLC). Within the first set, SAM was docked onto the active site of COMT using the crystal structure. However, because the algorithm only allows for flexible docking of the ligand and not side-chains, we also perform a second set of docking simulations using a structure of COMT derived from our SAM docking simulations. This structure contains the side-chain positions of SAM binding to apo-COMT as determined by MedusaDock, and thus we expect to recapitulate the same results.

Prior to all docking simulations, both the ligand and protein were prepared using LigPrep and the Protein Preparation Wizard workflow, respectively. Receptor grids were generated at the active site where SAM was occupied for each structure. The van der Waals scaling was kept at 1, with no additional constraints added to the protein or ligand. We performed docking simulations using the extra precision option and with post-docking minimization. Each SAM conformation derived from LigPrep was allowed to sample ring conversions and nitrogen inversions.

## Results and Discussion

### COMT Accommodates Ligands via an Induced-Fit Mechanism

**Figure 1:**
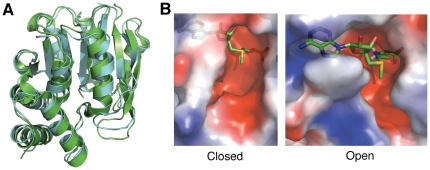
Structures of COMT open and closed conformations. (A) Structural alignment of closed COMT (generated from DMD simulations; shown in cyan) and open COMT (obtained from PDB: 3BWM; shown in green). The RMSD between the two structures is 1.518 Å. (B) Surface representations of the closed and open conformations of COMT (as labeled below each panel). Red surfaces of the protein correspond to negatively charged regions, blue surfaces correspond to positively charged regions, and the white surfaces are neutral. The conformation of SAM (depicted in green) is from the crystal structure and is placed for reference of comparison. The closed conformation of COMT does not allow SAM to bind and interact with the residues inside the active site, therefore creating a steric clash when superimposing SAM onto the closed COMT model.

Previous crystal structures of the rat COMT isoform show minor conformational differences between apo-COMT and holo-COMT [11]. Therefore, we performed DMD simulations of COMT in the absence of all substrates to determine whether the conformation of the human isoform remains identical to the crystal structure. An alignment of the crystal structure and simulated structure reveals few differences in the tertiary structure between the two with an overall root mean square deviation (RMSD) of 1.5 Å (Figure 1A). Secondary structural elements are completely retained in the absence of ligands. The most notable difference lies within the catechol-binding loop, which migrates closer to the active site in the absence of ligands.

Although the overall structure of COMT remains identical in the absence of ligands, the solvent accessibility of the active site changes (Figure 1B). In the crystal structure, COMT forms two pockets that accommodate the adenosine and methionine side-chains of SAM. The adenosine pocket partially collapses, and the methionine-binding motif completely closes in the absence of SAM. Comparison of the two active sites shows two main conformations of COMT: an open and a closed state. Within the closed state of COMT, SAM cannot bind inside the active site and therefore methylation cannot occur. To accommodate SAM, the COMT must initially open so that SAM can access the active site. Since only the adenosine-binding motif is partially open in the closed COMT structure, this region of the SAM cofactor may be responsible for initial binding and perhaps induces the open state. Because the closed COMT state makes the active site inaccessible, all docking simulations are performed with the open state of COMT under an assumption of induced fit. Within the open state, we refer to the absence of ligands as apo-COMT and the presence of all ligands as holo-COMT.

### Structural Characterization of SAM Conformations within Apo-COMT and Holo-COMT

**Figure 2:**
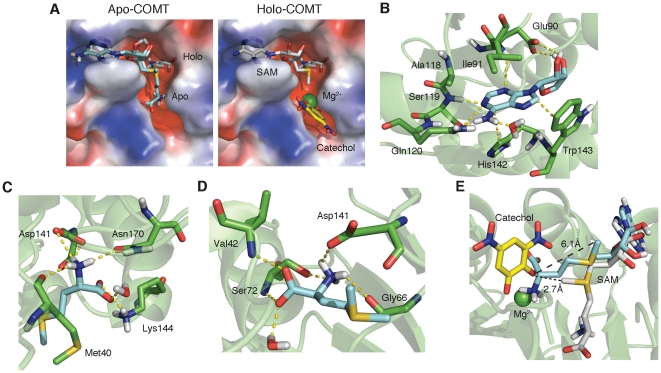
Binding configurations of SAM with and without metal. (A) Alignment of SAM poses with and without metal. Cyan structure represents without metal (apo-COMT; derived from simulation) and gray structure represents SAM in the presence of metal (holo-COMT; from PDB ID 3BWM). In the right panel, the yellow structure is dinitrocatechol and green sphere is Zn^2+^. Surface of protein is shown as an electrostatic map with blue regions representing clusters of positive charge and red representing regions of negative charge. (B) Contacts made with adenosine portion of SAM (identical for apo- and holo-COMT). Cyan structure is adenosine and green structures are corresponding residues. Yellow dashed lines indicate contacts. (C) Contacts made with methionine portion of SAM (apo-COMT). Cyan structure is methionine portion of SAM. (D) Contacts made with methionine portion of SAM (holo-COMT). (E) Comparison of angle between methyl donor and accepting hydroxyl in the presence and absence of metal. Cyan structure is apo-COMT and gray structure is holo-COMT.

We determine the binding poses of SAM in the absence and presence of a divalent cation metal by performing docking simulations within the open state of COMT using MedusaDock. In the absence of metal and substrate, portions of SAM bind to a different groove of COMT (Figure 2A). The adenosine moiety of SAM remains identical to the holo-COMT structure, with the Ile91 side-chain packing on top of the pyrimidine portion of adenine and the imidazole of His142 participating in a perpendicular edge-to-face aromatic interaction with the pyrimidine (Figure 2B). The imidazole of adenine participates in an additional edge-to-face interaction with the indole side-chain of Trp143. Additional polar contacts are made between residues 118-120 and the purine nitrogens. The two hydroxyl groups of the ribose participate in hydrogen bonding interactions with the side-chain of Glu90. A single water molecule from the active site satisfies an additional hydrogen bond requirement of the amine bound at C6 of the adenosine motif.

Similarities between the structures of SAM docked inside apo-COMT and holo-COMT end at the sulfonium center. In the apo-COMT complex, the terminal amine group of SAM is involved in a hydrogen-bonding network with the side-chains of Asp141 and Asn170 and the backbone carbonyl of Met40 (Figure 2C). The terminal carboxyl group participates in hydrogen bonding with Lys144 and a single water molecule. In the holo-COMT complex, the primary role of this water molecule is to occupy a coordination site of the divalent metal cation. Its secondary role is to form a hydrogen bond with the carboxyl terminus of the methionine side-chain (Figure 2D). In absence of the metal, satisfying the hydrogen bond of the carboxyl terminus becomes its primary role. Yet, its role is non-essential for the overall conformation of SAM in apo-COMT since Lys144 can satisfy both hydrogen bond requirements of the carboxyl terminus alone. The interactions highlighted here with methionine underlie an important point for why catalysis cannot occur without a metal. Lys144 is responsible for deprotonating the catechol to create the oxyanion responsible for attacking the methyl group. In this particular pose, the Lys144 is preoccupied in a hydrogen-bonding network and is unavailable to deprotonate.

Prior to catechol binding, a divalent metal cation must first displace the positively charged amine group. Several divalent cations are capable of contributing to enzymatic activity, although the native metal is magnesium *in vivo* [10]. Here we modeled the divalent metal cation using Zn^2+^, which is 80% as effective as magnesium. We find that upon metal binding, steric occlusion prevents the methionine of SAM from binding to the negatively charged pocket and is forced into an interior groove. The amine group of the methionine maintains a hydrogen bond with Asp141, albeit at a different position, but additionally forms hydrogen bonds with the backbone carbonyl of Gly66 and the side-chain of Ser72. The amide backbones of Ser72, Val42, and an additional water molecule form hydrogen bonds with the carboxyl group of SAM. Additional hydrophobic interactions are formed between the methionine side-chain and residues 40, 42, 60, 68, and 89 inside this pocket (Figure 2D).

The results found here are initially surprising because it is expected that SAM binds to apo-COMT as found in the crystal structure of the holoenzyme complex. The amine and carboxyl tail of SAM form favorable van der Waals contacts and satisfy their hydrogen bonds in both conformations. However, the carboxylate side-chains within the active site preferentially bind to the amine side-chain of SAM, as the polar contacts are stronger than those shared with the backbone carbonyl groups of holo-COMT.

**Figure 3:**
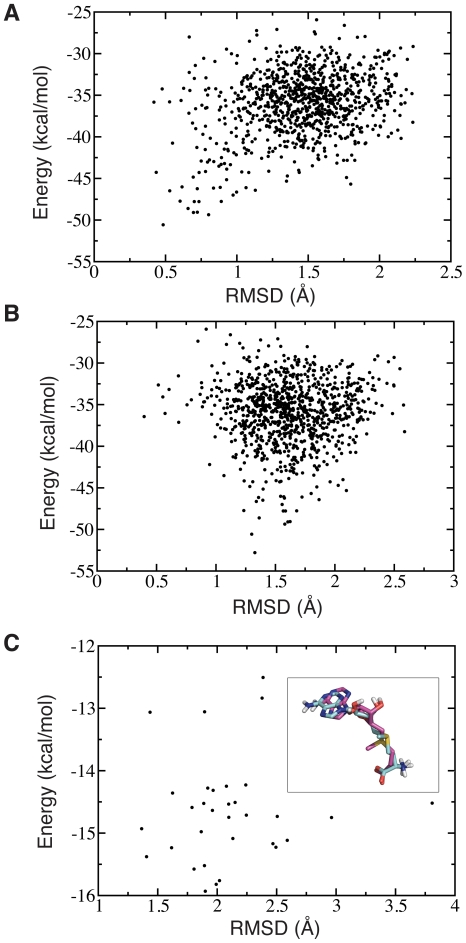
Analysis of SAM docking poses generated by MedusaDock and Glide. (A) Comparison of MedusaDock-generated structures of SAM versus the lowest energy pose of SAM obtained by MedusaDock. The lowest energy pose for SAM is considered the conformation that initially binds to apo-COMT. (B) Comparison of MedusaDock-generated structures of SAM versus the crystal structure of SAM within the holo-COMT complex. The lowest energy pose shown in this plot corresponds to the conformations depicted in Figure 2D,E. (C) Comparison of Glide generated structures of SAM versus the lowest energy pose of SAM obtained by MedusaDock. The inset shows an alignment between the lowest energy poses obtained by Glide (purple) and MedusaDock (cyan); RMSD  = 1.9 Å.

Most of the lowest energy docking poses generated for apo-COMT by MedusaDock are identical (Figure 3A) and deviate from the crystal structure (Figure 3B). However, the SAM conformation found for apo-COMT could potentially be an artifact of the MedusaDock/MedusaScore suite. To test the validity of our docking results, we utilize the docking program Glide (see Methods) to score the poses found for apo-COMT. In our initial docking test, we docked the crystal structure of COMT to SAM. Because Glide only allows flexibility to be modeled within the ligand, the lowest energy pose found was identical to the crystal structure (with an energy of −13.45 kcal/mol). We adjust for the lack of flexibility within the active site side-chains by redocking SAM to the MedusaDock-generated structure of COMT. Remarkably, we find that apo-COMT conformation of SAM is recapitulated with Glide (Figure 3C). Furthermore, this conformation is scored with a lower energy (−15.93 kcal/mol) compared to the crystal structure conformation.

### Role of Divalent Metal Cation in Methyltransferases

The role that magnesium plays in COMT activity has remained unclear to date. Without any metal bound to COMT, methylation is 6.2% as effective as compared to with all cofactors present [10]. Reports from the crystal structure suggest that Mg^2+^ is necessary for correctly aligning the oxyanion of the catechol substrate with the carbocation of SAM. However, it is unknown what the exact coordination complex of Mg^2+^ is *in vivo* due to the use of inhibitors in all holo-COMT crystal structures. These inhibitors usually contain electron-withdrawing groups on the catechol to lower the nucleophilicity of the reactive oxygen atom. Molecular dynamic simulations using a natural catechol substrate show catechol as a monodentate ligand of Mg^2+^ [12], [13]. The hydroxyl that coordinates with Mg^2+^ is a further topic of debate.

Here we suggest that a divalent metal cation may also be essential for structural rearrangements of the SAM cofactor. In our model, the orientation of the methionine without metal present positions the donating methyl group 6.1 Å away from the oxyanion, compared to a separation of 2.7 Å in the presence of metal (Figure 2E). Furthermore, the oxyanion and sulfonium no longer form a 180° angle in between the methyl group. This angle decreases to ∼45° in the absence of metal. Therefore the probability of SAM methylating the catechol is lowered.

Our results described here are derived purely from computation. Thus, crystallization of COMT in the absence of catechol substrate would be required to validate our mechanism. Two structures would be needed to support our mechanism: 1) COMT crystallized with SAM (or S-adenosyl-homocysteine) to show the alternate binding pose; 2) COMT crystallized with SAM and metal to show SAM in its holo-COMT conformation. Agreement between our docking poses and the proposed crystal structures would demonstrate the existence of this alternative SAM conformation in apo-COMT, and furthermore, show that the metal is required for proper SAM binding.

The mechanism proposed here is of broad biological interest since SAM serves as a common methyl donor for many methylation reactions, including CpG methylation [14]. The binding site of SAM on COMT is homologous with many SAM-dependent methyltransferase structures, with several unique residues that form part of the catechol-binding site. Thus, our mechanism could be applicable to a broad range of methyltransferases that require a divalent metal cation and SAM.
